# Acute gallbladder torsion - a continued pre-operative diagnostic dilemma

**DOI:** 10.1186/1749-7922-6-13

**Published:** 2011-04-13

**Authors:** Nicolas J Mouawad, Brianne Crofts, Rachel Streu, Randal Desrochers, Beth C Kimball

**Affiliations:** 1 Department of General Surgery, Saint Joseph Mercy Health System, Suite R-2111, PO Box 995, Ann Arbor, MI, 48106, USA

## Abstract

Acute gallbladder volvulus continues to remain a relatively uncommon process, manifesting itself usually during exploration for an acute surgical abdomen with a presumptive diagnosis of acute cholecystitis. The pathophysiology is that of mechanical organo-axial torsion along the gallbladder's longitudinal axis involving the cystic duct and cystic artery, and with a pre-requisite of local mesenteric redundancy. The demographic tendency is septua- and octo-genarians of the female sex, and its overall incidence is increasing, this being attributed to increasing life expectancy. We discuss two cases of elderly, fragile women presenting to the emergency department complaining of sudden onset right upper quadrant abdominal pain. Their subsequent evaluation suggested acute cholecystitis. Ultimately both were taken to the operating room where the correct diagnosis of gallbladder torsion was made. Pre-operative diagnosis continues to be a major challenge with only 4 cases reported in the literature diagnosed with pre-operative imaging; the remainder were found intra-operatively. Consequently, a delay in diagnosis can have devastating patient outcomes. Herein we propose a necessary high index of suspicion for gallbladder volvulus in the outlined patient demographic with symptoms and signs mimicking acute cholecystitis.

## Introduction

Acute gallbladder volvulus continues to remain a relatively uncommon process, manifesting itself usually during exploration for an acute surgical abdomen with a presumptive diagnosis of acute cholecystitis. The pathophysiology is that of mechanical organo-axial torsion along the gallbladder's longitudinal axis involving the cystic duct and cystic artery, and with a pre-requisite of local mesenteric redundancy. The demographic tendency is septua- and octo-genarians of the female sex, and its overall incidence is increasing, this being primarily attributed to increasing life expectancy. Despite significant challenges in pre-operative diagnosis, a high index of suspicion and prompt surgical intervention results in an overall mortality of approximately 5 percent.

### Case Report One

A 99-year-old Caucasian female presented with a 3 day history of acute onset right upper quadrant abdominal pain with intermittent radiation to the right flank and back. It was described as colicky in nature on a baseline dull character, and with no obvious precipitating, aggravating or relieving factors. Associated phenomena included anorexia and nausea, but no constitutional upset, vomiting, or change in bowel habit. Her medical history included peptic ulcer disease, uncontrolled hypertension, tobacco abuse, diverticulosis, a hiatal hernia, and dementia. Her surgical history was significant for an appendectomy.

Clinical physical examination revealed an apyrexic frail patient in no acute distress with stable vital signs. Focused abdominal examination demonstrated a soft, mildly distended abdomen with tenderness to palpation in the right upper quadrant, and a positive Murphy's sign. There was no overt peritonism. A reducible left inguinal hernia was also appreciated. Laboratory parameters yielded a mild leukocytosis with neutrophilia, and hypokalemia. Her liver function enzymes were elevated in a cholestatic distribution with a total bilirubin of 3.9 mg/dL, direct bilirubin of 0.9 mg/dL, and an alkaline phosphatase of 150 IU/L.

**Figure 1:**
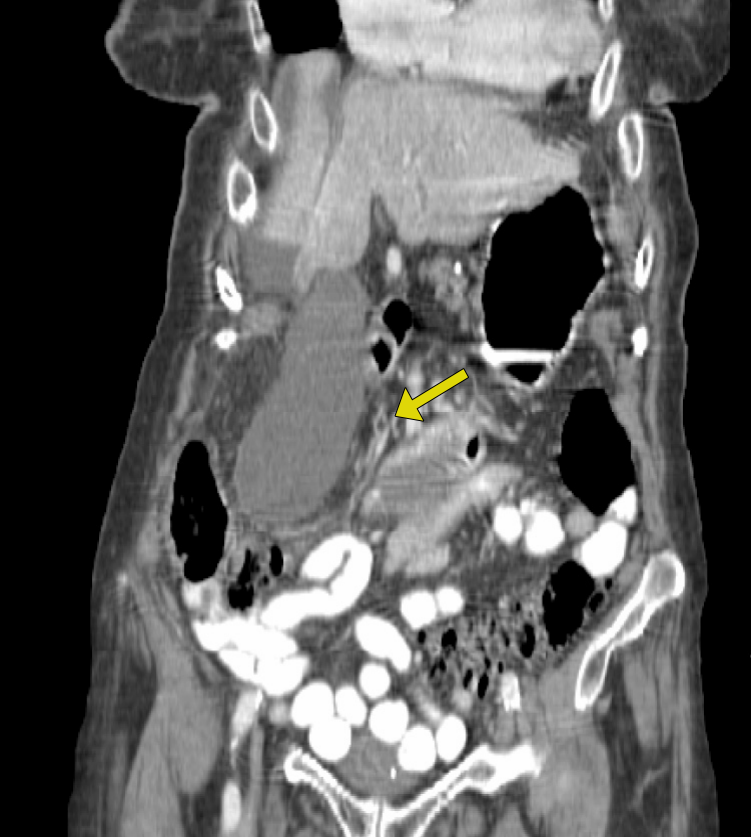
**Computed tomography scan in sagittal section demonstrating a large hydropic gallbladder**.

**Figure 2:**
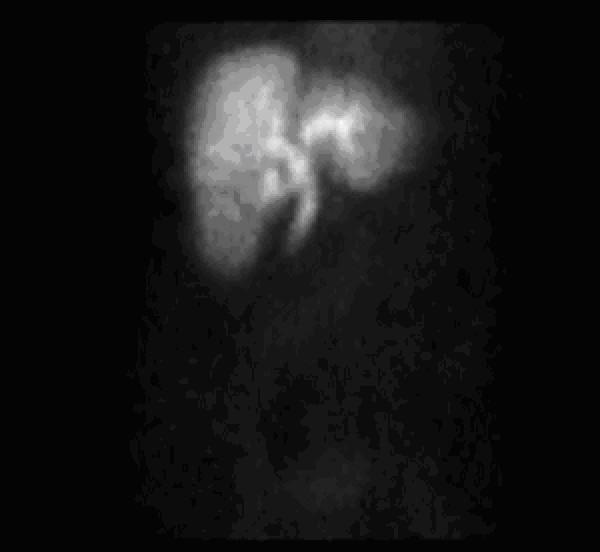
**HIDA scan in Patient 1 demonstrating uptake of tracer in liver without visualization of the gallbladder; delayed images showed excretion of tracer into the small bowel**.

A computed tomography (CT) scan was initially obtained prior to surgical consultation; it demonstrated a largely distended, hydropic gallbladder, pericholecystic fluid with wall thickening, a dilated common bile duct and prominent intra-hepatic bile ducts (Figure 1). A hydroxyiminodiacetic acid (HIDA) scan was then recommended that showed an uptake of tracer into the liver with excretion into the small bowel but without gallbladder filling (Figure 2).

**Figure 3:**
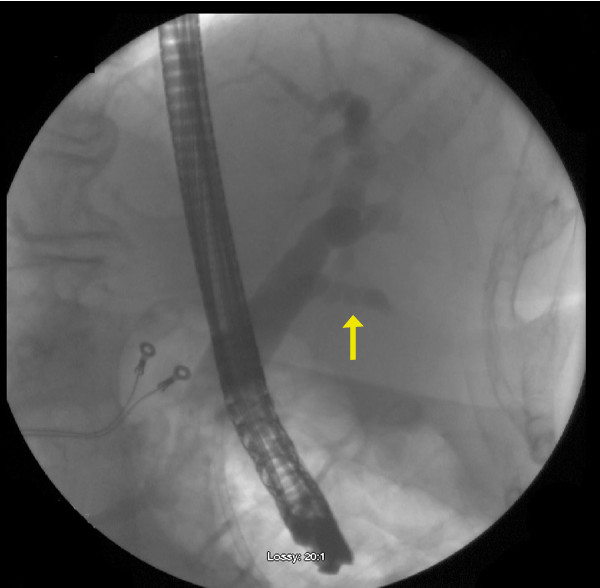
**ERC in Patient 1 showing mild dilatation of extrahepatic biliary tree with patent cystic duct (arrow) but without visualization of the gallbladder**.

The patient and her durable power of attorney (DPOA) refused the recommended surgical intervention of cholecystectomy. Non-operative measures with bowel rest, intravenous fluids and intravenous antibiotics were instituted. On hospital day 2, progressive elevation in her bilirubin and alkaline phosphatase prompted a gastro-enterology consultation and an endoscopic retrograde cholangiogram (ERC). This demonstrated dilatation of intra- and extra-hepatic bile ducts, a patent cystic duct, but non-filling of the gallbladder (Figure 3). A sphincterotomy was performed with evacuation of biliary sludge, but no stones were extracted; a common bile duct stent was placed. Her liver function tests did then trend towards normal.

A cholecystostomy tube was planned, but due to unfavorable anatomy through the liver, it could not be performed. On hospital day 6, despite a normal white blood cell count and apyrexia, she complained of worsening abdominal pain. Following an appropriate pre-operative cardiac workup, the patient and DPOA then consented to an open cholecystectomy with a presumptive diagnosis of acute cholecystitis.

**Figure 4:**
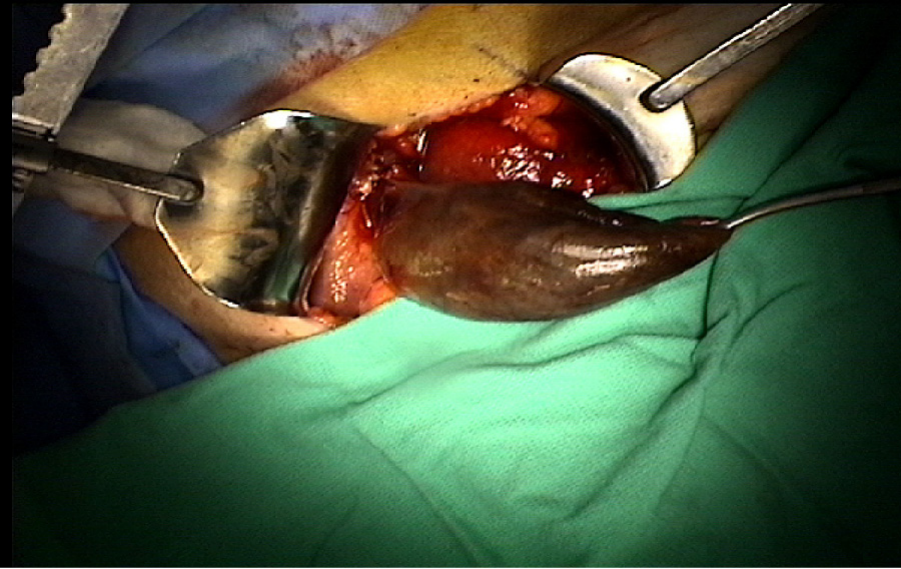
**Intraoperative finding**. Necrotic gallbladder twisted on its mesentery

On entering the abdominal cavity, a gangrenous distended gallbladder with omentum adhesed to it circumferentially was immediately noted (Figure 4). On further careful dissection, it was observed that the gallbladder was twisted on the cystic duct and artery, and the diagnosis of gallbladder volvulus was then made. The gallbladder torsion was reduced and a cholecystectomy was then performed in the usual fashion, with placement of a Jackson-Pratt drain in the gallbladder fossa. The specimen did not contain any gallstones. Histology revealed transmural necrosis consistent with volvulus.

She succumbed from cardio-respiratory failure on post-operative day 4, and was made comfort care respecting her do not resuscitate wishes.

### Case Report Two

An 89-year-old Caucasian female with no significant past medical history presented with acute right upper quadrant abdominal pain of approximately 5 hours duration. The pain radiated to the right flank, was crampy with intensities of sharpness, and was precipitated by a large meal. There were no aggravating or relieving factors. Associated phenomena included anorexia and nausea, but no fevers, chills or change in bowel habit. Her past surgical history was significant for an appendectomy.

**Figure 5:**
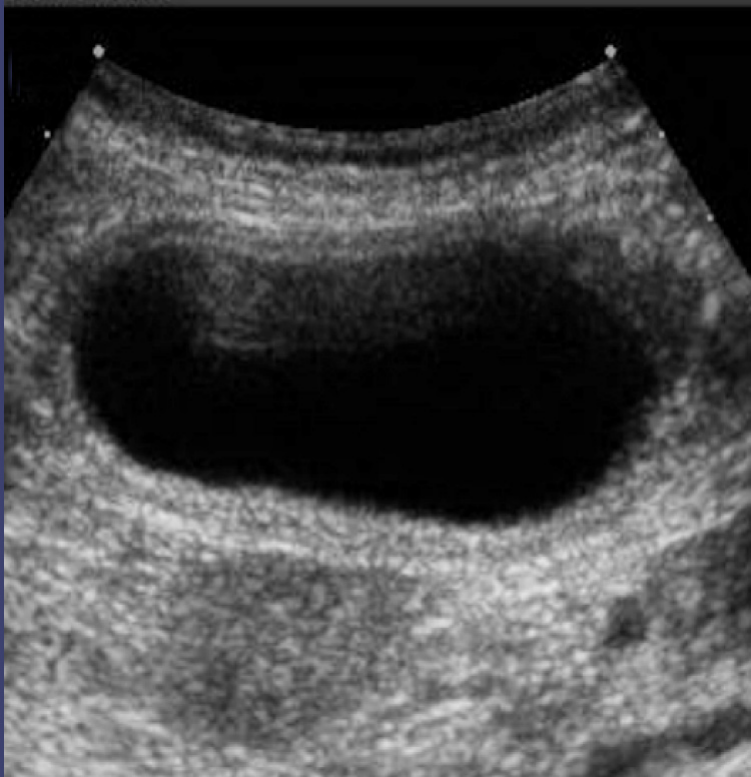
**The "floating gallbladder." **Ultrasound image in Patient 2 of a markedly enlarged gallbladder with a multi-layered hypoechoic rim demonstrating an edematous wall without calculi - the so-called classic description

**Figure 6:**
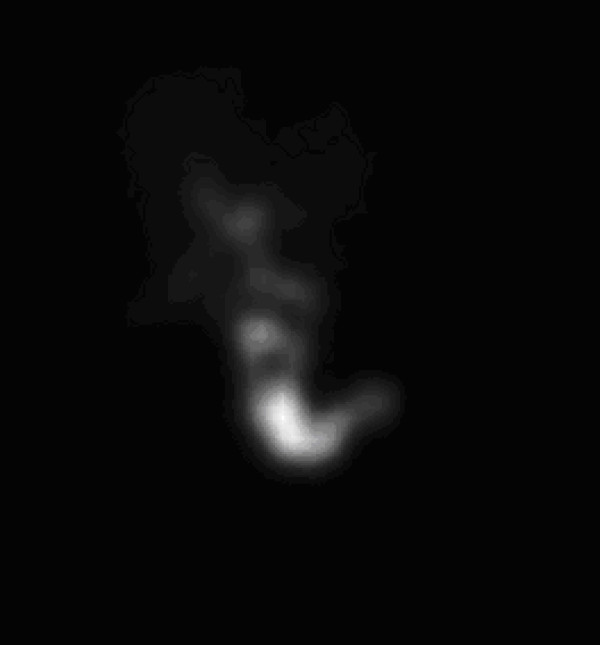
**HIDA scan in Patient 2 demonstrating non-filling of the gallbladder consistent with cystic duct obstruction**.

Focused clinical abdominal examination revealed a soft, mildly distended abdomen tender to palpation in the right hypochondrium; a positive Murphy's sign was present. She was afebrile with stable vital signs; laboratory parameters were within normal limits. An abdominal ultrasound revealed a distended gallbladder with mild wall thickening (Figure 5). There was no evidence of gallstones or biliary duct dilatation. A sonographic Murphy's sign was positive. A HIDA scan demonstrated non-filling of the gallbladder consistent with cystic duct obstruction (Figure 6).

After appropriate consent, the patient was taken to the operating room for a laparoscopic cholecystectomy with a pre-operative diagnosis of acute cholecystitis. After entering the peritoneal cavity and appropriate establishment of pneumoperitoneum, exploration quickly revealed an obvious necrotic gallbladder in the right upper quadrant. Further investigation noted that the gallbladder was twisted 180 degrees on its small pedicle with a thrombosed cystic artery. Following reduction of the torsion, the gallbladder was resected in the standard laparoscopic fashion. Histology demonstrated congested and ischemic serosa with necrotic mucosa consistent with torsion. Her post-operative course was unremarkable and she was discharged on post-operative day 1.

## Discussion

First reported by Wendel in 1898, and dubbed the "floating gallbladder", gallbladder volvulus is a recognized surgical entity [1]. It commonly affects women in their seventies and eighties, and the increased incidence of this condition may be attributable to increasing life expectancy. Despite its predilection for older ages, it has also been described in the pediatric population as early as 2 years of age [2].

Multiple hypotheses have been proposed as to the mechanism of gallbladder torsion, but the exact etiology continues to be unidentified. The pre-requisite of local mesenteric redundancy however is necessary for organo-axial torsion around its pedicle. Two anatomic variants have been described: 1) a torsion-prone mesentery, and 2) a mesentery supporting only the cystic duct allowing a completely peritonealized gallbladder to hang free. The susceptibility for rotational instability may be compounded by the elderly's fat loss and tissue atrophy suspending the gallbladder freely [3]. This was seen in both cases a probable precipitant for torsion.

**Figure 7:**
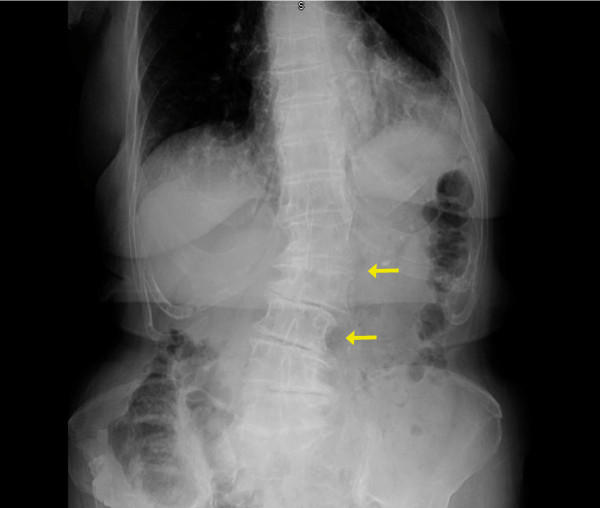
**Kyphoscoliosis of the spine in Patient 1 as a precipitant for gallbladder torsion**.

Further mechanisms may include violent peristaltic movements of neighboring organs, visceroptosis, and a tortuous atherosclerotic cystic artery [3]. Kyphoscoliosis of the spine has also been implicated as a fulcrum for torsion and was noted retrospectively in our first patient (Figure 7). An association of Saint's triad - the collection of diverticular disease, a hiatal hernia, and biliary pathology - has been previously reported by McAleese *et al; *this relationship may also be attributable to our first case when reviewing her history and to our knowledge, is the only other report of this association in the literature [4]. Nakao *et al *investigated 245 cases in the Japanese literature noting that cholelithiasis is an infrequent cause of gallbladder volvulus; gallstones were demonstrated in only a quarter of patients afflicted [5]. Their review proposed that an acute onset of abdominal pain with minimal episodes of fever or jaundice as well as a poor response to antibiotic use may be helpful in separating the diagnosis of torsion from cholecystitis albeit with a relative low accuracy [5].

Patients presenting to the emergency department with an acute surgical abdomen complaining of right upper quadrant abdominal pain invite a myriad of differentials including acute cholecystitis, choledochal cysts, choledocholithiasis, gastritis and peptic ulcer disease, intussusception, acute appendicitis, and nephrolithiasis. Laboratory parameters are equally unrewarding and non-specific noting general inflammatory changes.

The correct pre-operative diagnosis of gallbladder volvulus is very challenging, with less than a dozen cases having been diagnosed accurately with pre-operative imaging [3]. Despite technological advances in various imaging modalities, definitive diagnosis is generally achieved intra-operatively [6]. Historically, the classical finding seen on ultrasonography is that of a large, "floating gallbladder" that is exempt of stones. Other reports with computed tomography have noted an enlarged gallbladder that is outside of the gallbladder fossa, severe pericholecystic edema, and a prominent cystic artery to the right of the gallbladder [2,7,8]. This, however, continues to be relatively non-specific in clinical practice for intra-abdominal inflammation. Nuclear medicine scans with HIDA have been reported to demonstrate characteristic features pre-operatively [9]. It is, however, with magnetic resonance imaging (MRI) that accurate visualization of a twisted cystic duct has been shown, and may provide an optimal alternative for precise pre-operative diagnosis [10].

Operative surgical intervention involving reducing the torsion followed by removal of the gallbladder is the treatment of gallbladder volvulus. With further surgical advances, this has been reported safely with laparoscopic approaches in both the adult and pediatric population regardless of obtaining the correct diagnosis of torsion before surgery [10-12].

## Conclusions

Gallbladder volvulus continues to remain an uncommon surgical condition despite an increase in incidence. Although multiple imaging modalities are involved in attempting to obtain an accurate pre-operative diagnosis, no one has proven to be adequately sufficiently sensitive. The prompt diagnosis is critical to ensure that the patient undergoes an emergent index cholecystectomy rather than temporizing measures with antibiotics for a subsequent interval intervention. Herein we revisit and remind that the onus is on the surgeon to practice with a necessary high index of suspicion for gallbladder volvulus in the outlined patient demographic in order to circumvent treatment delays that may be fatal.

## Consent

Written informed consent was obtained from the patients for publication of these case reports and any accompanying images. A copy of the written consent is available for review by the Editor-in-Chief of this journal.

## Competing interests

The authors declare that they have no competing interests.

## Authors' contributions

NJM designed and drafted the manuscript, performed the literature search and was involved in the critical review. BC, RS, RD and BK were all involved in the peri-operative and surgical care of the patients. RD and BK provided supervision in drafting the manuscript and its critical review. All authors read and approved the final manuscript.
